# Daptomycin-Induced Eosinophilic Pneumonia: Can an Antibiotic Cause Pneumonia?

**DOI:** 10.7759/cureus.55298

**Published:** 2024-02-29

**Authors:** Subhan Saeed, Emaan Salam, Matthew Weaver, Sana Tahir, Aditya Bansal

**Affiliations:** 1 Internal Medicine, AtlantiCare Regional Medical Center, Atlantic City, USA; 2 Pulmonary and Critical Care, AtlantiCare Regional Medical Center, Atlantic City, USA

**Keywords:** daptomycin-induced eosinophilic pneumonia, daptomycin toxicity, eosinophilic infiltrates, daptomycin side effects, acute hypoxic respiratory failure

## Abstract

We present an interesting case of a patient who was discharged from the hospital on daptomycin and ertapenem in the setting of osteomyelitis. The patient did not have any respiratory symptoms during that hospital stay. A few weeks after discharge, the patient came back to the hospital with complaints of fever and shortness of breath. Chest X-ray showed pulmonary infiltrates. Initially, the patient was treated for acute respiratory distress syndrome (ARDS) vs pneumonia, but she did not improve. When labs showed significant eosinophilia, daptomycin-induced eosinophilic pneumonia became the working diagnosis, and the patient improved significantly when daptomycin was discontinued and steroids were started.

## Introduction

Daptomycin is a cyclic lipopeptide antibiotic that is used to treat different bacterial infections caused by Gram-positive bacteria, including methicillin-resistant Staphylococcus aureus (MRSA) and vancomycin-resistant enterococci (VRE) [1]. The use of daptomycin has increased recently as an alternative in vancomycin-resistant cases and so have the chances to explore the potential side effects [2] of this drug. Musculoskeletal adverse effects of daptomycin are known, but it can also have pulmonary adverse effects. Here, we report a case of a patient on daptomycin who came into the hospital with respiratory distress [3]. This case report addresses this rare side effect of eosinophilic pneumonia and its management in the inpatient setting as although it is rare but can be life-threatening if not treated appropriately.

## Case presentation

**Figure 1:**
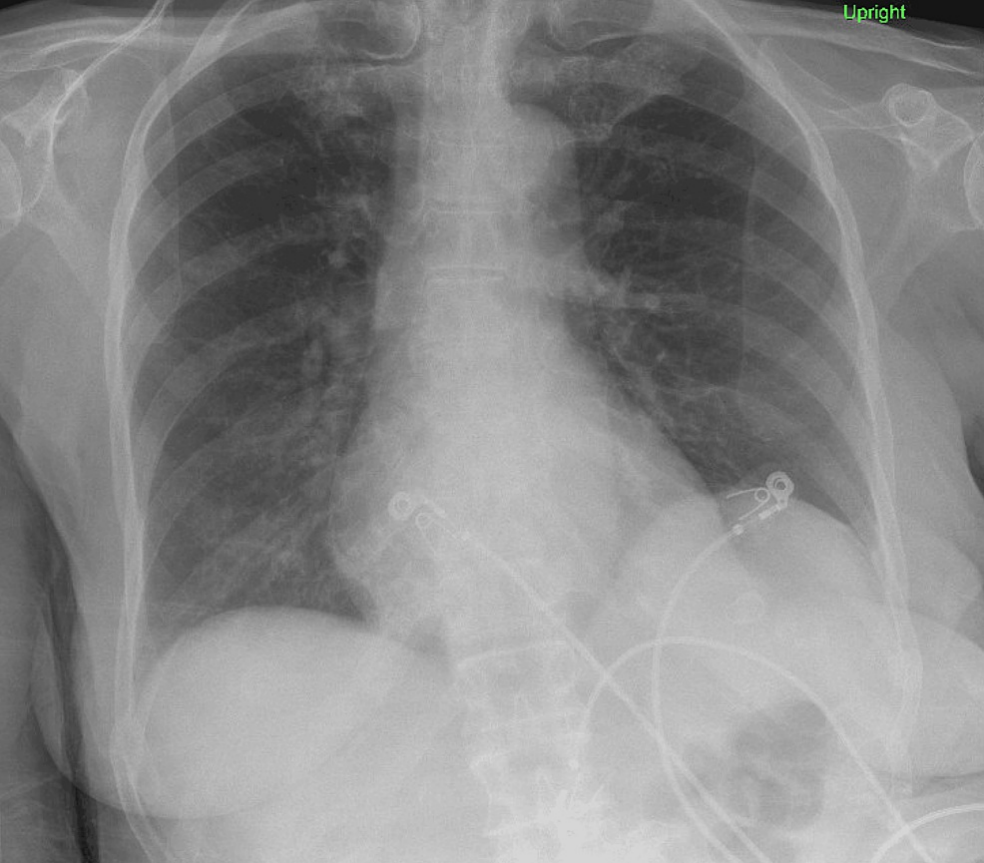
Chest X-ray before daptomycin use

Our patient is a 71-year-old female who presented to the emergency department from a rehabilitation facility with complaints of fever [4] and shortness of breath. She had been admitted to the hospital a month ago for osteomyelitis of the right foot and was discharged to a rehabilitation facility on daptomycin (500 mg IV daily) and ertapenem with a peripherally inserted central catheter (PICC) line in place. During that hospital visit, the patient's chest X-ray (Figure 1) was done, which did not show any infiltrates or consolidation at that time.

**Figure 2:**
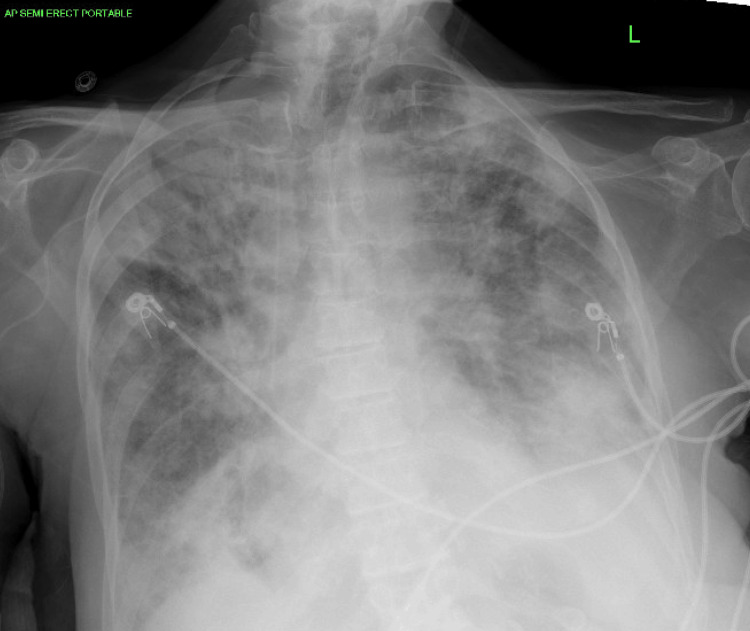
Chest X-ray after five weeks of daptomycin use

After a few weeks at the facility, the patient developed fever spikes, and a chest X-ray was done, which showed bilateral infiltrates. Levofloxacin was added to the ongoing antibiotics to cover for pneumonia. However, over the next several days, her fever persisted, and her breathing worsened prompting an ER visit. On presentation to the ER, the patient was hypoxic and tachypneic. Oxygen supplementation via nasal cannula was started. Chest auscultation revealed bilateral crackles. The initial working diagnosis was acute hypoxic respiratory failure secondary to early acute respiratory distress syndrome vs. healthcare-associated pneumonia. Meropenem was started with ongoing daptomycin, but symptoms did not improve. A chest X-ray in the hospital showed diffuse bilateral opacities (Figure 2) consistent with our initial diagnosis.

**Figure 3:**
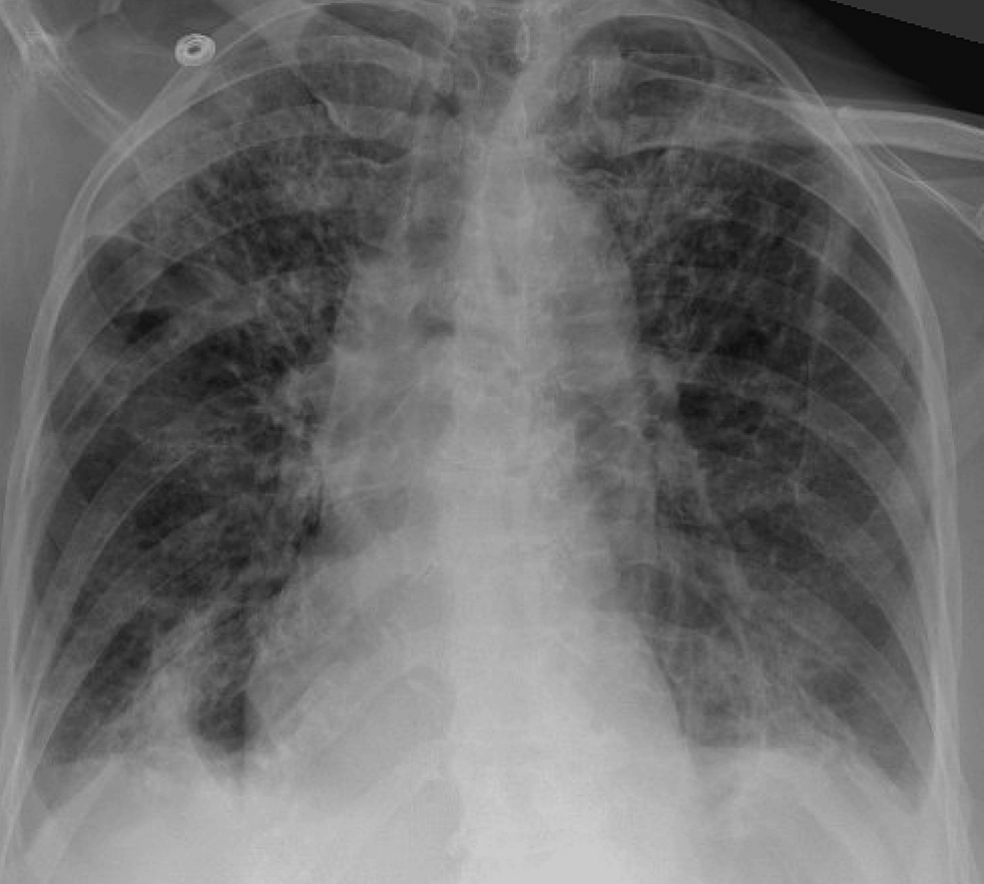
Chest X-ray six days after stopping daptomycin

However, when the differential blood count was obtained, it showed significant eosinophilia of greater than 1000. Considering that the patient is on daptomycin and the findings of eosinophilia and chest imaging, our final diagnosis was daptomycin-induced eosinophilic pneumonia [5,6]. Daptomycin was stopped and steroids were added. Soon after, the patient's oxygen requirement went down, fever subsided, and shortness of breath improved. Bronchoscopy for alveolar lavage was considered; however, this was withheld as she improved clinically. Repeat chest X-ray six days after discontinuing daptomycin showed significant resolution of bilateral pulmonary infiltrates (Figure 3). Eventually, after a few days, she was saturating well on room air and discharged on tapering doses of steroids, along with a different antibiotic regimen for her osteomyelitis. She was recommended outpatient pulmonary follow-up in two weeks.

## Discussion

With the recent increase in daptomycin use, it is important to know this potentially life-threatening adverse effect of daptomycin. There is a need to study the pathophysiology by which this drug causes eosinophilic pneumonia as it is not fully understood. The pathophysiology of acute eosinophilic pneumonia is thought to be caused by the detection of an antigen by alveolar macrophages, which leads to the recruitment of T-helper 2 lymphocytes and subsequent release of interleukin 5. Interleukin 5 promotes eosinophil production and migration to the lung [7,8]. More research and awareness of this risk is necessary so that the drug can be stopped promptly if pulmonary symptoms develop in a patient on daptomycin. For most patients who have suspected daptomycin-induced eosinophilic lung disease, a good history provides a presumptive diagnosis that can be confirmed by lung imaging and differential blood count, leading to early diagnosis and appropriate management. Furthermore, alveolar lavage has been done in previous case reports in order to establish a diagnosis, but most of the time, imaging and blood workup are enough; thus, we avoided an invasive procedure in our patient as she improved with our management [9].

## Conclusions

It is important to have a broad differential diagnosis in patients presenting with imaging findings of pulmonary infiltrates. As the pulmonary toxicity of daptomycin reverses with its discontinuation and administration of steroids, it would be important for physicians to have a low threshold to think of daptomycin causing eosinophilic pneumonia if a patient has a history of its use as it can reduce further complications and length of hospital stay. Failure to diagnose it at early stages can lead to respiratory failure. As the use of daptomycin is increasing, it is important for clinicians to diagnose and appropriately manage this rare but potentially life-threatening side effect.
